# Electrophysiological grading scale for polyneuropathy severity

**DOI:** 10.1371/journal.pone.0302491

**Published:** 2024-05-22

**Authors:** Alon Abraham, Vera Bril

**Affiliations:** 1 Neuromuscular Diseases Unit, Department of Neurology, Tel Aviv Sourasky Medical Center, Sackler Faculty of Medicine, Tel-Aviv University, Tel-Aviv, Israel; 2 Ellen and Martin Prosserman Centre for Neuromuscular Diseases, Division of Neurology, Department of Medicine, University Health Network, University of Toronto, Toronto, Canada; Sheikh Hasina National Institute of Burn & Plastic Surgery, BANGLADESH

## Abstract

**Objective:**

To establish a simple electrophysiological scale for patients with distal symmetric axonal polyneuropathy, in order to promote standardized and informative electrodiagnostic reporting, and understand the complex relationship between electrophysiological and clinical polyneuropathy severity.

**Methods:**

We included 76 patients with distal symmetric axonal polyneuropathy, from a cohort of 151 patients with polyneuropathy prospectively recruited from November 2016 to May 2017. Patients underwent nerve conduction studies (NCS), were evaluated by the Toronto Clinical Neuropathy Score (TCNS), and additional tests. The number of abnormal NCS parameters was determined, within the range of 0–4, considering low amplitude or conduction velocity in the sural and peroneal nerve.

**Results:**

Higher number of NCS abnormalities was associated with higher TCNS, indicating more severe polyneuropathy. Polyneuropathy severity per the TCNS was most frequently (63%-70%) mild in patients with a low (0–1) number of NCS abnormalities, and most frequently (57%-67%) severe in patients with a high number (3–4) of NCS abnormalities, while patients with an intermediate (2) number of NCS abnormalities showed mainly mild and moderate severity with equal distribution (40%).

**Conclusions:**

A simple NCS classification system can objectively grade polyneuropathy severity, although significant overlap exists especially at the intermediate range, underscoring the importance of clinical based scoring.

## Introduction

The combination of symptoms, signs, and electrodiagnostic findings is considered as the most accurate way to diagnose polyneuropathy [1], as the accuracy of the history and physical examination alone is limited [2]. Similarly, the Toronto Consensus Panel on Diabetic Neuropathy requires the presence of an abnormal nerve conduction study (NCS) or other objective measure in order to confirm the presence of polyneuropathy, while symptoms and / or signs enable the diagnosis of only possible or probable polyneuropathy [3].

In addition to enhancing diagnostic accuracy and providing objective confirmation for the presence of polyneuropathy, electrodiagnostic assessment results may correlate with polyneuropathy severity. Baba et al. has previously published a diagnostic and staging algorithm for diabetic polyneuropathy based on NCS [4], that was subsequently adapted/translated by Himeno et al. [5]. Previous study has shown that diabetic polyneuropathy severity is consistently related to diabetic control [6]. Severity staging scales were also published for mononeuropathies due to entrapment syndromes including carpal tunnel syndrome [7], ulnar neuropathy at the elbow [8], and for tarsal tunnel syndrome [9]. Grading polyneuropathy severity based on NCS may provide additional important objective clinical information besides confirming the diagnosis, and promote standardized and more informative electrodiagnostic reporting, as there are currently no widely accepted guidelines regarding reporting of polyneuropathy severity.

In the current study, we aimed to establish a polyneuropathy severity grading scale in patients with distal symmetric axonal polyneuropathy, which is the most commonly encountered polyneuropathy, and understand the complex relationship between electrophysiological and clinical polyneuropathy severity.

In contrast to Baba’s classification [4], which was based on sural and tibial NCS results in patients with diabetic polyneuropathy, we aimed to explore the utility of sural and peroneal NCS, which are considered as the most sensitive for diagnosing polyneuropathy [1] in patients with non-diabetic polyneuropathy, and to define 3 instead of 5 severity scales, based on polyneuropathy severity as captured by the Toronto Clinical Neuropathy Score (TCNS) [10].

## Materials and methods

Patients attending the Prosserman Family Neuromuscular Clinic, Toronto General Hospital, University Health Network, were prospectively recruited from November 2016 to May 2017, as a part of a study aiming to explore the performance of the TCNS in non-diabetic polyneuropathies. The Research Ethics Board of the University Health Network approved the study protocol and all patients provided written informed consent prior to participation [11].

151 Patients were diagnosed with non-diabetic polyneuropathy using the England and Toronto consensus criteria, based on clinical and NCS results, small nerve fiber tests, or with a well-defined clinical setting such as onset during chemotherapy [11]. Patients with prediabetic polyneuropathy were also included in the study, although considered by some as part of the spectrum of diabetic polyneuropathy. In order to maintain relative homogeneity, 76 patients with distal symmetric axonal polyneuropathy were included, based on compatible clinical presentation, as well as clinical and electrophysiological examination results, while patients with other polyneuropathies, such as genetic, demyelinating, or vasculitic polyneuropathy were excluded due to their unique clinical phenotypes. In addition, patients older than 75 years old were also excluded, as some consider absent sural response as normal at this age [12]. The various etiologies for polyneuropathy in the current study included idiopathic (46), prediabetes (16), chemotherapy induced (8), B12 deficiency (3), MGUS (2), chronic renal failure (2), alcohol related (2), medication induced (1), and critical illness (1) (few patients had more than one etiology).

Patients underwent NCS according to the protocols of the Toronto General Hospital (University Health Network) electrophysiology laboratory, including motor responses from the median and peroneal nerves, and sensory responses from the median and sural nerves. The number of abnormal NCS parameters was determined for each patient, within the range of 0–4. Abnormal values were considered as low amplitude or conduction velocity in the sural and peroneal nerve as follows: sural sensory nerve action potentials (SNAP) amplitude < 6 μV in patients <60 years old (<3 μV at age 60–70, and <1 μV at age 70–75 years old [12]), peroneal compound muscle action potential (CMAP) amplitude < 2 mV, and sural or peroneal conduction velocity < 40 m/s. For example, a 65 year old patient with a low sural amplitude (e.g. 1.5 μV) and low sural sensory conduction velocity (e.g. 35 m/s), with normal peroneal CMAP amplitude (e.g. 2.5 mV), and normal peroneal motor conduction velocity (e.g. 45 m/s), will have a score of 2 abnormal NCS parameters.

As compared with other nerves, the median nerve is much more prone to entrapment due to other reasons except polyneuropathy, therefore abnormalities in this nerve were not included in the current scale. Small fiber tests included Laser Doppler flare imaging and quantitative thermal threshold testing.

In addition, patients underwent routine clinical evaluation, and were evaluated by the TCNS [10] and functional scales, using the Neuro-QoLTM (Quality of Life in Neurological Disorders) (http://www.neuroqol.org/Pages/default.aspx). Additional testing included vibration perception thresholds (VPT) using the method of limits and grip strength. More detailed description of clinical and ancillary tests can be found elsewhere [11]. All data are in the S1 File.

### Statistical analysis

Data were analyzed using the statistical package for social sciences (SPSS) software version 27. Comparisons of demographics, various polyneuropathy etiologies, electrophysiological findings, and clinical scales between groups were performed using the Kruskal–Wallis one-way analysis of variance for continuous variables, and χ2-test for categorical variables. Correlation between the number of NCS abnormalities and the TCNS was determined using spearman correlation coefficient. P values <0.05 were considered as significant.

## Results

**Fig 1:**
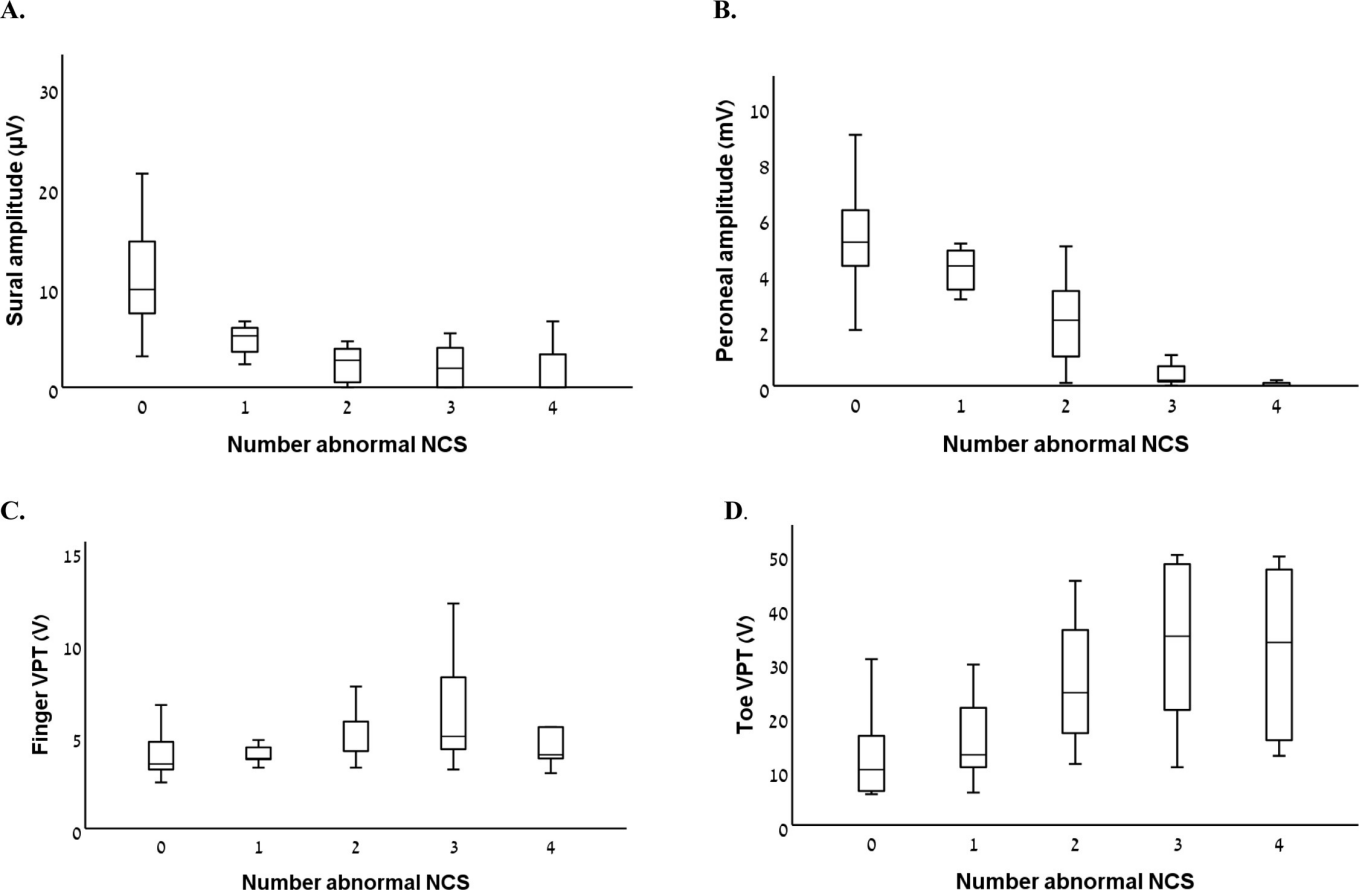
Selected nerve conduction study (NCS) parameters and vibration perception thresholds (VPT) according to the number of abnormal NCS parameters.

76 patients with a mean age of 55 (±11) years, including 43.4% females, were included. A higher number of NCS abnormalities at the sural and peroneal nerves was associated with worse median sensory SNAP amplitude and sensory and motor conduction velocities, but not with median motor distal latency and CMAP amplitude (Table 1 and Fig 1). In addition, a higher number of NCS abnormalities was associated with higher VPT at the toe, but not at the finger, higher TCNS, and a lower score for lower limb function (Table 2 and Fig 1).

**Table 1:** Comparisons of demographics and electrophysiological measures in 76 patients with polyneuropathy according to the number of abnormal nerve conduction study parameters.

# of abnormalities:	0	1	2	3	4	Total	p-value
N	37	16	10	7	6	76	-
Age mean (SD)	54.3 (11.3)	53.8 (10.2)	56.2 (10.3)	54.1 (7.9)	60.7 (10.8)	55 (11)	0.69
Female sex n (%)	54.1	50	20	28.6	16.7	43.4	0.17
**NCS:**							
Sensory:						Norms	p-value
Median Amp (μV)	65.5 (39)	40.4 (34.3)	42.1 (33.9)	30 (22.5)	35.7 (19.7)	≥20	0.04
CV (m/s)	56.9 (8.2)	52.7 (8.9)	49.8 (9.2)	48.2 (10.7)	54.2 (5.2)	≥50	0.07
Sural Amp (μV)	11.2 (5.5)	5 (2.1)	4 (5.2)	3.4 (1.6)	1.6 (2.8)	≥6	<0.001
CV (m/s)	47.4 (4)	45.3 (4.3)	28.2 (20)	39 (4.5)	12.3 (19.1)	≥40	<0.001
Motor:							
Median Amp (mV)	9.9 (3.2)	9.7 (3.1)	8.2 (4.2)	8.4 (1.6)	7.8 (3.7)	≥5	0.41
DL (ms)	3.3 (0.6)	3.5 (0.6)	3.8 (0.9)	3.8 (0.4)	3.6 (0.3)	<4.5	0.11
CV (m/s)	56.3 (3.2)	55.5 (4.1)	52 (3.2)	48.4 (2.7)	49.7 (2)	≥50	<0.001
Peroneal Amp (mV)	5.4 (1.8)	4 (1.6)	2.6 (1.5)	1.3 (1.7)	1 (0.1)	≥2	<0.001
DL (ms)	4 (0.6)	4.1 (0.6)	5 (0.9)	5 (0.7)	7.8 (4.7)	<6.6	<0.001
CV (m/s)	45.9 (3.2)	44.6 (4.1)	36.3 (13.3)	29 (13.5)	9.8 (15.6)	≥40	<0.001

Data presented as mean (standard deviation). NCS ‐ Nerve conduction study; Amp- Amplitude (in mV for motor and μV for sensory nerves); DL–Distal Latency; CV–Conduction Velocity.

**Table 2:** Comparisons of clinical measures in 76 patients with polyneuropathy according to the number of abnormal nerve conduction study parameters.

# of abnormalities:	0	1	2	3	4	Norms	p-value
N	37	16	10	7	6	**-**	-
**VPT:**							
Finger (V)	4 (1.4)	4.2 (0.9)	4.6 (1.2)	4.6 (1)	5.8 (4)	<5	0.18
Toe (V)	13.8 (9.4)	16.7 (8.2)	24.7 (10.9)	26 (13.9)	31.9 (17.2)	<15	<0.001
**Grip**							
Right (Kg)	32.3 (12.5)	31.2 (14.5)	32.3 (12.2)	30.7 (12.7)	34.3 (10.7)	-	0.99
**Function:**							
Upper limb	93 (13)	90.9 (15.2)	92.3 (11.7)	84.3 (22.1)	92 (12)	100	0.69
Lower Limb	82.3 (15.2)	80.9 (15.3)	83.8 (14.4)	65.1 (24.9)	64.2 (20)	95	0.03
**TCNS:**							
Symptoms	2.8 (1.3)	3.8 (1.6)	3 (1.6)	3.1 (1.8)	3.7 (0.5)	0	0.19
Sensory deficits	1.7 (1.7)	2.3 (1.9)	2.8 (1.8)	2.9 (1.7)	3.8 (1.2)	0	0.04
Reflexes	1.6 (2.6)	1.8 (2.5)	2.8 (3)	5.6 (2.8)	5 (3.3)	0	0.002
Total	6.1 (4.1)	7.9 (4.2)	8.6 (5.3)	11.6 (4.6)	12.5 (3.5)	0	0.002

Data presented as mean (standard deviation). VPT ‐ Vibration perception threshold; TCNS ‐ Toronto Clinical Neuropathy Score.

Mean TCNS fell within the range of mild neuropathy (<8) for 0–1 NCS abnormalities: 6.1 (±4.1) and 7.9 (±4.2) correspondingly, slightly below the range of moderate neuropathy (9–11) for 2 abnormalities: 8.6 (±5.3), and within the range of severe neuropathy (>11) for 3–4 abnormalities: 11.6 (±4.6) and 12.5 (±3.5) correspondingly (Table 2).

**Fig 2:**
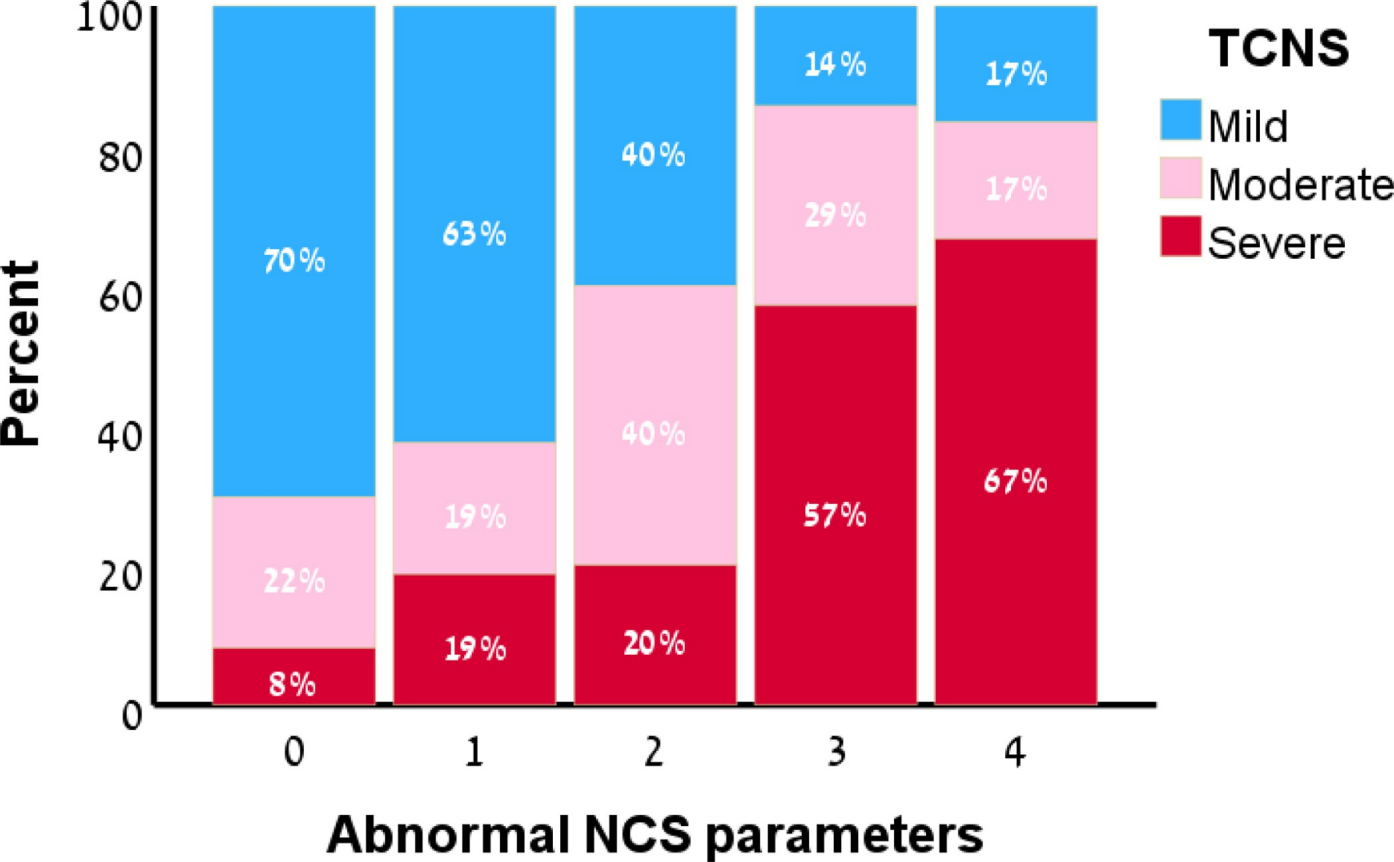
Percent and number of patients with different TCNS scores, according to the number of abnormal nerve conduction study parameters. TCNS ‐ Toronto Clinical Neuropathy Score; NCS ‐ Nerve condition study.

Polyneuropathy severity per the TCNS was most frequently (63–70%) mild in patients with a low number of NCS abnormalities (0–1), and most frequently (57–67%) severe in patients with a high number of NCS abnormalities (3–4), while patients with an intermediate number of NCS abnormalities (2) showed similar distribution between mild (40%), and moderate (40%) polyneuropathy (Fig 2). Grading polyneuropathy severity by the number of NCS abnormalities was therefore set as 0–1 for mild, 2 for moderate (or mild-moderate), and 3–4 for severe. The number of abnormalities correlated moderately with the TCNS (r = 0.50, p< 0.001).

## Discussion

Electrophysiological assessment constitutes an important part of the evaluation of patients with polyneuropathy, serving as an objective tool in order to confirm the presence of polyneuropathy [1–3]. The current study results suggest that electrophysiological assessment may have an added value enabling objective grading of polyneuropathy severity. Specifically, we found that the number of abnormal parameters in NCS performed on two lower limb nerves (sural and peroneal), is associated with polyneuropathy severity, as measured by the TCNS, although with significant overlap, mainly at the intermediate range. In addition, the number of abnormal parameters was associated with higher VPT at the toe (indicating greater sensory loss), and more disability as measured by a lower limb function scale, further supporting the correlation between polyneuropathy severity and NCS results. As distal symmetric polyneuropathy affects mainly the lower limbs, it is not surprising that there were no differences between subgroups regarding upper limb findings.

While a low or high number of abnormalities was associated with a mild or a severe polyneuropathy respectively in most cases (57–70%), an intermediate number of abnormalities was associated with moderate polyneuropathy in only 40% of patients. As additional 40% had mild polyneuropathy, an intermediate range might be considered more appropriately as indicative for mild to moderate polyneuropathy. These results suggest that at the extremes, the number of abnormalities performs better and might be more useful than in the intermediate range, which needs to be taken in account when used in the clinical setting. Nonetheless, overall, in most cases our grading scale fitted the polyneuropathy severity based on a validated clinical scale.

In contrast to Baba’s NCS classification [4], which is based on sural and tibial nerve assessment in diabetic polyneuropathy, we used the sural and peroneal nerves, as they are considered as the most sensitive for diagnosing polyneuropathy [1]. The choice of nerve can be influenced by cultural factors with some societies preferring tibial to peroneal. Our grading system is also simpler to use in the clinic, as we defined only 3 grades instead of 5 grades used by Baba’s classification [4], did not include F-waves, and defined motor amplitude as normal or abnormal, instead of defining three different levels of abnormalities based on tibial CMAP amplitude. In addition, while Baba’s classification was based on diabetic polyneuropathy, we studied only non-diabetic polyneuropathy, although we did include patients with prediabetic polyneuropathy, considered by some as part of the spectrum of diabetic polyneuropathy, suggesting that NCS scoring can be accomplished in all patients with polyneuropathy, regardless of etiology.

Our study has several limitations. The number of patients is our study is limited, especially within the subgroup with severe polyneuropathy. In addition, in order to determine polyneuropathy severity we used a single scale ‐ the TCNS [10], although numerous other scales are available. However, the TCNS [10], was found to be valid in multiple studies, including in non-diabetic polyneuropathy [11].

In conclusion, we suggest a simple electrophysiological classification system for objective grading of polyneuropathy that may promote standardized and more informative electrodiagnostic reporting, although significant overlap exists, necessitating a comprehensive clinical evaluation before determining polyneuropathy severity.

## Supporting information

S1 File SPSS file with study data. (SAV)
